# Genome Sequence of Clinical Strain Pseudomonas aeruginosa NRD619

**DOI:** 10.1128/MRA.01013-20

**Published:** 2020-10-29

**Authors:** Laura Sisk-Hackworth, Andrew Sue, Saima Aslam, Dwayne Roach

**Affiliations:** a Department of Biology, San Diego State University, San Diego, California, USA; b Division of Infectious Diseases and Global Public Health, Department of Medicine, University of California San Diego, La Jolla, California, USA; Loyola University Chicago

## Abstract

Here, we report the complete genome sequence of the multidrug-resistant (MDR) strain Pseudomonas aeruginosa NRD619, assembled via long- and short-read hybrid assembly. P. aeruginosa is a Gram-negative bacterial pathogen that is a significant public health burden. NRD619 was isolated from a left ventricular assist device (LVAD) draining sinus tract.

## ANNOUNCEMENT

Pseudomonas aeruginosa is a Gram-negative bacillus commonly found in soil and water as well as plants and humans. Classified as an opportunistic pathogen, P. aeruginosa is a major cause of illness and mortality in humans. Importantly, persistent infections arise in patients with immunosuppressive or chronic illnesses, such as cystic fibrosis, burns, wounds, cancer, and conditions requiring ventricular assist devices (1, 2). The genome of P. aeruginosa, which is especially large for a bacterium and averages 5.5 to 7 million base pairs (Mbp), has provided an understanding of the metabolic and pathogenic mechanisms that underlie the success of this versatile pathogen (2). It is also a model for understanding microbial evolution in chronic diseases, particularly those strains with increased antibiotic resistance (3).

In this study, we present the complete genome sequence of P. aeruginosa NRD619. This strain was isolated from a draining sinus tract and from blood cultures from a left ventricular assist device (LVAD) recipient after almost 2 years of ongoing infection. Blood was collected in BD Bactec blood culture tubes, and 5 ml was incubated at 37°C in aerobic and anaerobic environments. Positive blood cultures and a swab culture from the draining sinus tract were then streaked onto MacConkey, Columbia nalidixic acid (CNA), and chocolate agar plates and incubated at 37°C. Final identification was performed using matrix-assisted laser desorption ionization–time of flight (MALDI-TOF) mass spectrometry. Although LVADs have revolutionized the treatment of advanced heart failure, infections remain a substantial risk (1). The strain exhibited antibiotic sensitivity to amikacin (<8 μg/ml), aztreonam (<2 μg/ml), ceftazidime (4 μg/ml), gentamicin (<2 μg/ml), piperacillin-tazobactam (8/4 μg/ml), and tobramycin (<2 μg/ml) and resistance to cefepime (16 μg/ml), ciprofloxacin (>2 μg/ml), and meropenem (8 μg/ml). This patient was treated under institutional review board (IRB)-approved protocol 191417, which included sample collection.

Chromosomal DNA was extracted from an overnight culture from a single colony on Luria broth (LB) agar at 37°C using the NucleoSpin microbial DNA isolation kit (Macherey-Nagel, Düren, Germany). The DNA Link Sequencing Lab (San Diego, CA) prepared a library using the SMRTbell template prep kit version 1.0, and fragments smaller than 20 kb were removed with the automated BluePippin size selection system (Sage Science, Beverly, MA). After validation using the Agilent 2100 Bioanalyzer instrument, the library was sequenced on 1 single-molecule real-time (SMRT) cell of the PacBio RS II platform (Menlo Park, CA). A total of 171,246 long reads and 1,647,640,675 bp were produced, corresponding to an estimated coverage of 255-fold. After subread filtering and *de novo* hierarchical genome assembly (HGAP version 2.3) (4), consensus polishing with Quiver resulted in a single contig of 6,461,122 bp. The *N*_50_ value for the PacBio subreads was 13,055 bp, and after polishing, the *N*_50_ value for the assembly was 6,461,122 bp. Unless otherwise noted, default parameters were used for all software tools. Next, the Microbial Genome Sequencing Center (MiGs; Pittsburgh, PA) prepared a library for Illumina NextSeq 550 paired-end sequencing using the Nextera DNA Flex sample preparation kit (Illumina, San Diego, CA, USA). A total of 4.6 million reads of 150 bases were obtained. The paired-end reads were processed with fastp version 0.19.4 (5) and used to polish the long-read HGAP assembly using Pilon version 1.23 (6). The complete genome sequence of P. aeruginosa NRD619 is 6,438,945 bp with a rich G+C content of 66.42%. This is consistent with other P. aeruginosa genome sequences (sizes, 5.5 to 7 Mbp; G+C contents, 65 to 67%) (7).

Next, we annotated the complete genome of NRD619 using RAST (Rapid Annotations using Subsystems Technology) (8). This estimated 6,296 coding regions, revealing a wide range of metabolic and pathogenic mechanisms, including choline transport (*choV*, *choW*, *choX*, and *betT1*) (9), growth regulation (*potA*, *potB*, *potC*, *potD*, *potF*, *potG*, and *potH*) (10), and metabolic (*madL* and *madM*) (11) and transcriptional regulator (*pcaR*) (11, 12) genes. In addition, NRD619 contains genes encoding multiple drug efflux pump systems (MexA-MexB-OprM, MexC-MexD-OprJ, MexE-MexF-OprN, and MexX-MexY-OprM), antibiotic resistance (C and D beta-lactamases), and biofilm formation (*pelG*) (13).

P. aeruginosa NRD619 was found to harbor 2 prophages as predicted using PhiSpy (14). One prophage is 22,692 bp long and has 95% homology to the *Pseudomonas* phage phi3, while the other is 41,439 bp, with 95% homology to the *Pseudomonas* phage MD8. Interestingly, the latter MD8-like prophage also contains a gene with homology to the *luxR* transcriptional regulator that is involved in quorum sensing (15).

In summary, we report the new genome sequence of a P. aeruginosa isolate that caused chronic infection in an LVAD patient. This new genome of P. aeruginosa NRD619 displays features of virulence and antibiotic resistance, such as a bacteriocin, multidrug efflux pumps, and beta-lactamases, which are common in human isolates of P. aeruginosa (16, 17). Given the high rate of MDR infections, a greater understanding of metabolism, virulence, resistance, and evolutionary mechanisms will aid in improving treatment and developing alternative antimicrobials like phage therapy (18, 19).

### Data availability.

The genome sequence of P. aeruginosa strain NRD619 was deposited in GenBank under the accession number CP060703, where PGAP annotation is also available. RAST annotation of the genome is available on Zenodo (https://zenodo.org/record/4062504#.X5G_B3V7nIU). Reads were deposited in the Sequence Read Archive under accession numbers SRX8974486 for PacBio reads and SRX8974485 for Illumina reads under the BioProject accession number PRJNA658200 for BioSample number SAMN15860813.
